# Experiences in the Philippine Islands3Read at the 27th annual meeting of the Alumni Association, Buffalo University Medical College, May 2, 1902.

**Published:** 1902-09

**Authors:** W. P. Kendall

**Affiliations:** Surgeon U. S. Army, Fort Porter, N.Y.


### Experiences in the Philippine Islands.[3]

By MAJOR W. P. KENDALL, M. D.,
Surgeon U. S. Army.

AFTER listening to the able paper by my confrère, it would be superfluous for me to deal with the subject of the effects produced by the modern weapon. In consequence I shall eliminate it from what I have to say, as far as possible, and touch upon it only where it has a direct relation to the consideration of some particular case I shall present.

Instead, I will invite your attention to conditions in general, which, because of their influence upon any given injury, must be

1. Tessier. Cited in American Year Book of Medicine and Surgery, 1897, p. 35.

2. Thos. F. Harrington, Journal of Tuberculosis, Vol. XI., p. 5.

3. Read at the 27th annual meeting of the Alumni Association, Buffalo University Medical College, May 2, 1902.

considered as important factors when studying the probable outcome as a whole. The few cases which I shall present owe their interest more to the fact that they are somewhat unusual rather than that they can be called wonderful. Remarkable surgery is more apt to be seen in your hospitals which, to say the least, are as well supplied as are those who are working in the field.

A great commander has said that an army fights upon its belly. It is as essential that the surgeon should have a foundation upon which to work, as it is that troops should be properly and well fed. The vitality which is apparent, when you see a regiment of young, lusty fellows drawing out from your station is not present months afterward in the Philippine Islands.

The exposures to a constant, though not extreme elevation of temperature, caused by a tropical sun; to days of marching through bamboo forests, over roads, or paths rather, which would stall a horse, with their bottomless depths of clayey mud; to nights of waking while lying in troughs of stagnant water or pouring rain, nourished only by such food, possibly for days, as in addition to the 30 or 40 lbs. of his equipment, a man is able to carry upon his back; to say nothing of poisoned drinking water, and swarming mosquitoes, are not potent factors, either in the upbuilding or maintaining of a condition desirable for operative procedures.

In all military operations these things exist, they are a part of it, and unavoidable. While always to be considered they exist in the Philippine Islands to an extreme degree, furnishing food for thought for both commanding general and his surgeon.

The condition though insidious in its approach and immeasurable in its extent is, nevertheless, constantly present and becomes a prominent factor every time a patient is brought to the operating table. As applied to any given case it may not be considered in any sense vital; it is, however, a factor and has an immediate bearing upon every gunshot injury, even though unrecognised. I mention it in order to call your attention, *en passant*, to that which, while dealing with the more direct and tangible, might be lost to view.

As disease is to be classed among the first of these influences which break the backbone of an army, even while the *prima viæ* is well supplied, let us glance for a moment at some of those which more particularly engage the attention of the army surgeon in the Philippine Islands.

Mosquitoes are everywhere, the year round, though not, as a rule, in numbers that would be considered excessive; the clay soil, holding as it does, the large rain fall, and the abundant vegetation furnish exactly the required conditions for their birth and development. All unprotected sooner or later become infected with malaria, and while the variety is not so severe in its nature as that found in Cuba and Puerto Rico, still the worst forms are not unknown, and the best is bad enough to eventually undermine an entire regiment. It is one of the most important diseases the army in the Philippines has to contend with.

The next most important is probably dysentery, of which two varieties are seen. The acute, not very troublesome, and the chonic or amebic. Of this latter, cases are to be found everywhere, and unfortunately once infected, no one can see the end of it. It is too apt to be a lingering sickness covering months and years, with its ups and downs, until death finally ends the scene. So far, no good remedy has been discovered, though they do improve when sent to a colder climate. All the usual remedies are absolute failures. High rectal injections of aqueous solutions of quinine sulphate, of a strength of from 1-1000 to 1-2000 are most in vogue, and seem to have some effect, but real cures are widely separated.

Typhoid fever exists there, as it does here, and apparently runs about the same course. The population is dense. No attempt is made at sanitation, and the water, which is obtained from wells, or wherever it can be found, runs filth, so the wonder is small that non-immunes quickly fall victims. The natives probably owe their immunity to an illustration of the survival of the fittest, the weaker having died early.

Owing to the fact that vaccination has been little practised, smallpox is found generally affecting the children, the elders having had it. The troops have been slightly troubled with it and a few deaths have occurred.

Tuberculosis is said to be a very frequent and fatal malady among the natives, but as yet it has no marked influence upon the health of the army.

Beriberi and leprosy exist almost altogether among the natives or Chinese. The moving of the camps to a dry, sunny spot has terminated outbreaks of the former; while a Lazaretto on some suitable island is contemplated for the 30,000 lepers now in the islands. It is not necessary to state that the American forces have not been troubled with either of these diseases though it is true, that a few cases of beriberi have been seen. Holding, as I think most do, with Manson, that the poison of beriberi is an ether emanating from a microbe whose habitat is the moist worm soil, instead of the human being, the nitrogenous starvation theory as a cause, is abandoned and while not neglecting good food, the one object seems to be, to get these patients away from the infected spot; the results following such steps warrant the entertainment of Manson's theory.

While I was there the bubonic plague broke out, but was confined, almost altogether to the native population and Chinese. If it were not for the abominable way in which these people live, crowding together as they do by the dozen, there would be no difficulty in handling the disease.

Venereal troubles always play a large part in the health of an army of occupation. It was said, with what truth I do not know, that there was less in the country before our arrival, than afterward. There appeared to be about the usual amount.

One can hardly dismiss the thought of infected water without touching upon the diseases produced by the presence of intestinal parasites, and while every variety known to the profession was occasionally seen, that known as ankylostomosis, or Egyptian chlorosis, and caused by the presence of the ankylostomum duodenale, is the most harmful. As these cases are, until in advanced stage, all ambulating, it is impossible to estimate their numbers. In Puerto Rico it is said that more than 84 per cent. of all the natives sick and well, harbor these parasites; it is not unfair to believe the disease is more widely spread than we realise. The other parasitical diseases are easily handled; but this one, though thymol at times is effective, is not at all controlable.

Both typhoid fever and amebic dysentery, were greatly diminished in numbers wherever they were able to get a pure water supply, or could boil that which was available. The anophelės works only after about sunset, consequently protection for the troops at night was indicated, and mosquito bars were furnished when possible. Could this be thoroughly done, practical immunity might be expected.

It will be remarked that all the common diseases of these islands are such as can be readily controled by sanitary measures, and the fact is that nothing but careful attention to these principles is necessary to make of the Philippine Islands as healthy a spot as is that of Buffalo.

In dealing with the subject of gunshot wounds, having considered the general conditions which might influence the course of any given injury, one immediately thinks of the direct factors, such as the nature of the missile, the rapidity of its flight, its size, and the like, together with the portion of the body injured, whether fleshy, bony, vascular or tendinous, whether the head be struck, or the abdomen penetrated; if a vital part has not been struck, that which is of more importance than all the rest put together, is the immediate treatment the wound receives.

As a general rule bullet wounds are aseptic, and cases were frequently met which proved its accuracy. We all know why an aseptic wound does not remain so. On the battle field conditions are not such as are found in a modern, well-regulated operating room. If accidents happen to prevent the working out of carefully arranged plans, it was but the inevitable, the reverse of what was expected. What else is war anyway? Somebody's plan is upset. If it were necessary to dress a wound with a bandage which had been carried by the man himself for days, exposed to rain and dust, and saturated with the perspiration of his body, what must be the consequence?

If suppuration follow is it any wonder? The doctor, if one were present, may have worked to the tune of whistling bullets; often, generally the case. The nearest, even impure, water may have been within the enemy's line; possibly he was in a hurry. It is not impossible that he had many to dress. This does not affect the rule. It does emphasize the fact that every possible precaution must be taken at the first dressing. In support of the statement, a few cases came under my observation. A ball (probably mauser) passed from side to side through the knee joint, and beneath the patella. The wound healed without incident, and in a few days the man was as well as ever.

A lieutenant was shot in the right side, the ball passing from before backward, probably traversing the liver. In ten days the man was up and around and a short time afterward reported for duty.

A captain was struck in the thigh, fracturing the femur about three inches above the knee-joint. He was dressed on the field; carried thirteen miles in an ambulance; put upon a Hodgson splint and beyond a severe synovitis, had no complications, and although the ball remained in situ, made the normal recovery of a simple fracture.

I am able to present to you one of these cases, that of a man who received a ball in the left thorax, which, ranging directly backward, and lodging beneath the scapula, did great injury to vessels and nerves, but suppuration did not follow and he brought the ball home with him, to have it removed here in your hospital.

Similar cases might be cited by anyone who has seen many. While cases which have become infected are daily occurrences. Because of their severity let me cite three which had became infected, and unfortunately by the bacillus ærogenes capsulatus. One of these was a flesh wound of the calf of the leg, the other two, flesh wounds of the thigh and buttocks. Such dressings that were at hand were used; and in all these an attempt had been made to arrest slight hemorrhage by introducing into the wound gauze, or oakum. All three reached the hospital, and all died within a few hours, due to the terrible depressing effects of the toxin produced by this organism, the injuries in themselves being absolutely nothing. How these wounds became infected it is, of course, useless to surmise, unless it were possible that the dust of battle had settled, either upon the dressings or into the wound. Two of these came from the same field, and it is probable that, like the tetanus bacillus, this organism finds its habitat in the soil of certain localities. There are few pathogenic bacteriæ whose presence the system can so poorly support; however, the effects produced by all are bad, and the deductions are plain. Don't infect a gunshot wound. If you are not in a position to dress it aseptically, do only so much as is necessary to protect it while in transit to a point where it can be properly attended to.

Recognising the necessities, an attempt has been made to accomplish this for those wounded upon the battle field, and today every United States soldier carries what is termed an "emergency packet;" this consists of sterilised gauze with bandages wrapped in waxed paper; when new, these answer the purpose admirably, and the soldier, or comrade, always has at hand a suitable dressing. The trouble is, however, that after a while they become infected by saturation of either rain, or perspiration from the man's body, in consequence losing their virtue.

In examining a gunshot wound, the question at once arises as to the course of the ball. Usually there is no difficulty in determining it. The rule that the wound of entrance is small while that of exit is larger, is almost always demonstrated.

Cases are, however, not wanting when the most careful examination fails to disclose the direction the ball has taken. You would imagine the man himself could help you, but due to the excitement of battle, or the fact that the attack came from different directions, this is not always the case; fortunately, it seldom is a matter of moment. Of one thing you can be certain, the extent of the injury between the two points is always greater than would be judged by an examination of the openings in the integument; frequently the amount of damage done is amazing, and that when but two round clean cut holes exist in the skin. This is due particularly in the soft tissues, to what is known as the explosive effect of bullets. When a ball is fired into a can of water, instead of merely passing through it, as might be expected, the can is exploded in all directions, following the physical law that pressure upon liquids is the same in all directions. It is believed that the fluids contained in the tissues act as that in the can, and the consequence is, that the effect produced by the passage of a foreign body is transmitted widely, destroying the tissues nearest to its track, while at the same time producing a devitalising effect upon those more remote. This rule applies alone to the soft tissues. Bones resist this force unless struck when the force becomes transmitted through another medium. In the case of bones, force is transmitted in such a way as to produce trouble at points some distance from the track of the missile. This consists in either shattering or splintering. The greater destruction being done, as would be expected, by the larger ball; while the smaller may even bore its way through a bone, causing no other damage than is produced by this small gimlet-like track. The difference in the effect produced by the old Springfield with its 45 caliber ball, traveling with an initial velocity of 1,100 feet per second, as compared with the modern Mauser, or Krag-Jorgenson, with its 30 caliber, traveling about 1,900 feet per second, may be summed up in a general way at about 50 per cent. in favor of the larger.

A comparison of the records of the Surgeon-General's reports for the civil war and the late ones shows that of all men wounded during the former, about 18 per cent. were killed outright, as compared with only 12 per cent. so killed during the Spanish-American and Philippine wars.

In every gunshot injury, especially with the ball remaining in situ, the question at once arises, as to how much shall be done; whether we shall begin a possibly hopeless search for a deeply buried ball, or whether we shall cleanse it thoroughly and wait. The case of the captain with a broken femur, illustrates that the latter can be done, and I believe that it should be in all cases where there is a probability of non-infection. Of course, if it has become infected, the sooner it is thoroughly dressed and the ball removed the better. Surgery is a synonym for assistance. It is certainly better to have an aseptic compound fracture of the femur even though there be present some fragments and a ball, rather than a condition we can imagine, brought about by superfluous handling. It is true the case I have mentioned is the exception; they too frequently were infected. I believe, however, it illustrates a principle and furnishes a standard we should hope for and strive to reach. If there be an exception, it is in the case of penetrating wounds of the abdomen. While we can take a chance for our patient and find out whether the wound be infected or not in other wounds of the body and extremities, it is here a doubtful policy. A possible perforation of the intestine means too much and, although by so doing, I take a stand upon the wrong side in the light of some statistics from the reports of recent scenes of action, I cannot accept the statement that there are more recoveries from gunshot wounds of the abdomen where operative procedures were not attempted, than where they were. This is but an arraignment of ourselves. We kill our patient. As no one is prepared to accept this, what explanation can be given?

Mine is, that there are two classes of cases entirely incomparable. Among those operated upon, with death following, were all the severe cases. Those in whom the injury was great, with possibly several perforations of the intestine, they demanded an operation and would have died anyway, they were not made worse by the operation; while among those who were not operated upon, yet recovered, were all those whose injuries were slight, with no perforation; properly operated upon, they would have recovered, as they did; they, too, would not have been made worse by the operation.

Unfortunately, I am unable to point to any of my own cases in proof of my position. Others have been more fortunate, and we can certainly hope for it, even when we do not realise it. I had the opportunity to operate two or three times, always several hours after the receipt of the injury, and as I have said, I was not successful. I do not believe they would have recovered left to themselves. On the other hand, I saw two cases which were examples of the second, or milder class. One was operated upon, the other was not; both recovered, and both would have recovered if left to themselves.

In one, the ball entered at the seventh costal cartilage, upon the left side, ranged downward and backward, and lodged beneath the integument of the left lumbar region; having in its course entered the peritoneal cavity, perforated the omentum, and gone out again, at a point some 2½ inches from where it entered, without in anyway doing injury to the intestine; in the other the ball entered at a point 1 inch above and the same distance inward from the left ant. sup. spinous process of the ilium, going I know not where, but it came out at the gluteal fold of the opposite side, near the tip of the cocyx. One was operated upon because it was seen early and because it was considered to be imperative; the other was not operated upon because he was not seen until late, when it was certain that he was in no danger. Both cases illustrate what a ball may do, and one at least illustrates why it may not always be necessary to operate. In this latter case, the intestine surely was not damaged. Though how it can be possible, I believe the same to have been true of the former, I should be pleased to have some one offer an explanation. One thing I have not mentioned, and that is that the small ball traveling rapidly, is less liable to be deflected than is the large one traveling slowly. It is not possible that the ball in this case could have followed any fascia.

Speaking of apparently fatal wounds, recalls one of a man who received the ball in the third intercostal space of the left side, at the border of the sternum; its point of exit was directly behind at the base of the spine of the scapula. As 400 c.c. of blood were drawn from the left pleural sac, it certainly passed through that; the rest I leave to your imagination. He made an uneventful recovery with no sequelæ.

As an apparent illustration of the great length of time certain pathogenic organisms can lie dormant in the system, I recall the case of an officer who had had an abscess, the result of a lacerated wound of the lower end of the left femur, when ten years of age. The recovery was apparently complete, no symptoms having been noticed since that time, and during his twenty-seven years of service, he had been on sick report but three days. With no other discoverable cause, an acute osteomyelitis of the femur developed, and ran a rapid course. The pathologist reported a pure culture of the staphylococcus pyogenes aureus. If there were a connection between the two, this organism had lain dormant forty-two years.

While upon an independent marauding expedition, a man received a bolo wound, at the upper part of the right parietal region. At the dressing no fracture was detected; five days later, when I first saw him, he was unconscious; had a temperature of 105° and was having frequent, irregular convulsions. There was no paralysis, and the pupils, which reacted somewhat to light were about normal. When placed upon the table and the wound opened, it was found that the lateral halves of the skull moved easily, one upon the other. Trephining was done and a large blood clot and spiculæ turned out. He made an uninterrupted recovery. In a few hours, apparent consciousness returned. In thirty-six hours all convulsive movements had ceased, and in forty-eight, the temperature reached 100°. It might seem as though he had had enough, but the court added five years.

One case, while it presented no complications, yet gave us a great reputation among the native population, was that of a Filipino woman, for whom we successfully removed an ovarian cyst, which weighed 40 lbs. Those of you who recall the size of those women who were at the exposition last summer will see no great difference between the size of the tumor and the woman. They don't operate in those cases as early as you do.

FORT PORTER, N. Y.

